# Heterochromatic Genome Stability Requires Regulators of Histone H3 K9 Methylation

**DOI:** 10.1371/journal.pgen.1000435

**Published:** 2009-03-27

**Authors:** Jamy C. Peng, Gary H. Karpen

**Affiliations:** Lawrence Berkeley National Laboratory, Department of Genome and Computational Biology, Berkeley, California, United States of America; Department of Molecular and Cell Biology, University of California at Berkeley, Berkeley, California, United States of America; Stowers Institute for Medical Research, United States of America

## Abstract

Heterochromatin contains many repetitive DNA elements and few protein-encoding genes, yet it is essential for chromosome organization and inheritance. Here, we show that *Drosophila* that lack the Su(var)3-9 H3K9 methyltransferase display significantly elevated frequencies of spontaneous DNA damage in heterochromatin, in both somatic and germ-line cells. Accumulated DNA damage in these mutants correlates with chromosomal defects, such as translocations and loss of heterozygosity. DNA repair and mitotic checkpoints are also activated in mutant animals and are required for their viability. Similar effects of lower magnitude were observed in animals that lack the RNA interference pathway component Dcr2. These results suggest that the H3K9 methylation and RNAi pathways ensure heterochromatin stability.

## Author Summary

DNA damage from the environment is very common in animal cells, yet most of the time they are repaired efficiently and the integrity of the hereditary material is maintained. The genomes of most eukaryotes, such as humans and fruitflies, contain repeated DNAs that pose major challenges to genome stability. For example, recombination between repeated DNAs can result in chromosome rearrangements, a hallmark of cancer and birth defects. Repeated DNAs are contained within the part of the genome known as heterochromatin, which is characterized by a special type of chromatin packaging not prevalent in the rest of the genome, known as euchromatin. We use cytological and genetic analyses of the fruitfly model organism and demonstrate that the chromatin structure of heterochromatin plays a key role in efficient DNA damage detection and/or repair of repeated DNAs. Loss of a key chromatin modification (methylation of histone H3 at lysine 9) leads to increased spontaneous DNA damage specific to heterochromatin and activation of DNA repair cell cycle checkpoints. If left unrepaired, these DNA breaks cause cellular and organismal death. Remarkably, human euchromatin and fruitfly heterochromatin share similar physical features, such as repeated DNA content, intron lengths, and open reading frame sizes. Understanding heterochromatic DNA damage repair will yield critical information about how mammalian genome stability is maintained and how defects in these processes may contribute to human disease.

## Introduction

The eukaryotic genome is partitioned into cytologically and functionally distinct heterochromatin and euchromatin. Heterochromatin was initially defined as the part of the genome that remains compacted throughout the cell cycle and stains intensively with DNA dyes [1]. Heterochromatin is rich in tandemly repeated sequences and transposable elements [2], and as a result was mistakenly assumed to be genomic ‘junk’ with no function. In fact, heterochromatin contains essential protein coding genes [3], and encodes indispensable chromosomal functions such as centromeres, telomeres, nuclear organization, and meiotic homolog pairing [4]–[7].

Different chromatin states have been correlated with patterns of post-translational histone modifications. Chromatin associated with actively expressed genes contains methylated lysine 4 of histone H3 (H3K4me) and hyper-acetylated histones. In contrast, H3K9me2 and me3 modifications in ‘silent’ chromatin have become a standard characteristic of heterochromatin [8]. Recent studies have shown that RNA interference (RNAi) pathways are required for the initial recruitment of H3K9 methyltransferases (HMTases), such as *clr4* in *S. pombe* and *Su(var)3-9* in *Drosophila*, and for the establishment and maintenance of heterochromatin [9],[10].

In addition to regulating functions such as transcription and chromosome organization, chromatin is involved in the cellular response to DNA damage, especially double-stranded breaks (DSBs) [11]. The chromatin structure around DSBs assists recruitment and retention of DNA repair components and cell cycle checkpoint proteins. For example, phosphorylated H2A variants—serine 139 of H2Ax in mammals, serine 129 of H2A in yeast (γH2AX in mammals), and serine 137 of H2Av (γH2Av) in *Drosophila*—are important for recruitment of cohesins [12], ATP-dependent chromatin remodelers and various DNA repair factors [13],[14]. Dephosphorylation of H2A variants serves as a signal for cell cycle checkpoint recovery [15]. Other histone modifications implicated in the DNA damage response are phosphorylation, acetylation, and methylation of histone H4 residues, H3K79 methylation, H2BK123 ubiquitination, and H2AS129 phosphorylation [16].

The two main repair responses to DSBs are homologous recombination (HR) and non-homologous end joining (NHEJ). Comparative studies in *S. cerevisiae*, mammals and *Drosophila* showed many similarities while highlighting significant differences [17]. For example, a mutation in Rad51, a critical component of the HR pathway, causes lethality in mouse but does not impact viability in *S. cerevisiae* or *Drosophila* [18]. Compared to other systems, DNA repair factors in *Drosophila* appear to be highly redundant, and repair pathways can functionally compensate for each other [19].

Differences between organisms also exist in the DNA damage checkpoint pathways, which delay cell cycle progression to facilitate efficient DNA repair. The main regulators are the phosphoinositol kinases ATM and ATR in mammalian and yeast systems, and ATR/*mei-41* in *Drosophila* [20]–[22]. ATM and ATR recruitment to DSBs results in H2Av phosphorylation and activation of checkpoint kinase 1 (Chk1, *grp* in *Drosophila*) and/or checkpoint kinase 2 (Chk2, *lok* in *Drosophila*), which then delay cell cycle progression until the damage is repaired [20],[23],[24]. Chk1- and/or Chk2- mediated phosphorylation in response to DNA damage regulates the G1-S transition, S phase progression, G2-M transitions, and the metaphase-anaphase transition [25]–[27]. ATM (*tefu*) in *Drosophila* functions in telomere protection in addition to apoptotic signaling via the p53 pathway (Oikemus et al., 2004). In contrast to mammalian systems, p53 in *Drosophila* does not directly participate in the DNA damage checkpoint response, and instead activates the apoptosis pathway in response to persistent, unrepaired DNA damage [28].

Chromatin functions in the DNA damage response provokes questions about whether the distinct compositions of euchromatin and heterochromatin impact DNA integrity. Replication across repeated sequences can result in sequence alterations and replication fork stalling and collapse, which can lead to DSBs. In addition, recombination between homologous tandem and dispersed repeats alters repeat lengths and generates exchange between non-homologous chromosomes, which can cause translocations and aneuploidy [29]. These challenges suggest that heterochromatic DNAs may utilize different regulatory mechanisms during replication, repair, and recombination to ensure genome stability. This hypothesis is further supported by the observations that heterochromatin is consistently replicated later in S phase than euchromatin [30], and that reciprocal meiotic recombination does not occur in heterochromatin [31]–[33].

We previously showed that animals mutated for proteins that regulate heterochromatin structure (*Su(var)*s), including the H3K9 HMTase *Su(var)3-9*, the H3K9me binding protein Heterochromatin Protein 1 (*HP1*), and components of the RNA interference (RNAi) pathway, contained significantly increased levels of extrachromosomal repeated DNAs [34]. This phenotype led us to hypothesize that these pathways may regulate additional aspects of genome stability in repeated DNAs and heterochromatin. Here we show that compromised heterochromatin composition, specifically due to mutations in *Su(var)3-9* and the *dcr-2* siRNA pathway component, results in increased spontaneous DNA damage in heterochromatin in somatic and meiotic cells. Detailed analyses of *Su(var)3-9* mutants showed that diploid cells exhibit chromosomal defects such as translocations and aneuploidy. In addition, activation of DNA repair and mitotic checkpoints is required for cellular and organismal viability of mutant animals. We conclude that the H3K9 methylation and RNAi pathways are required to ensure the general stability of heterochromatic sequences.

## Results

### Heterochromatic DNA damage is increased in *Su(var)3-9* mutant somatic cells

**Figure 1:**
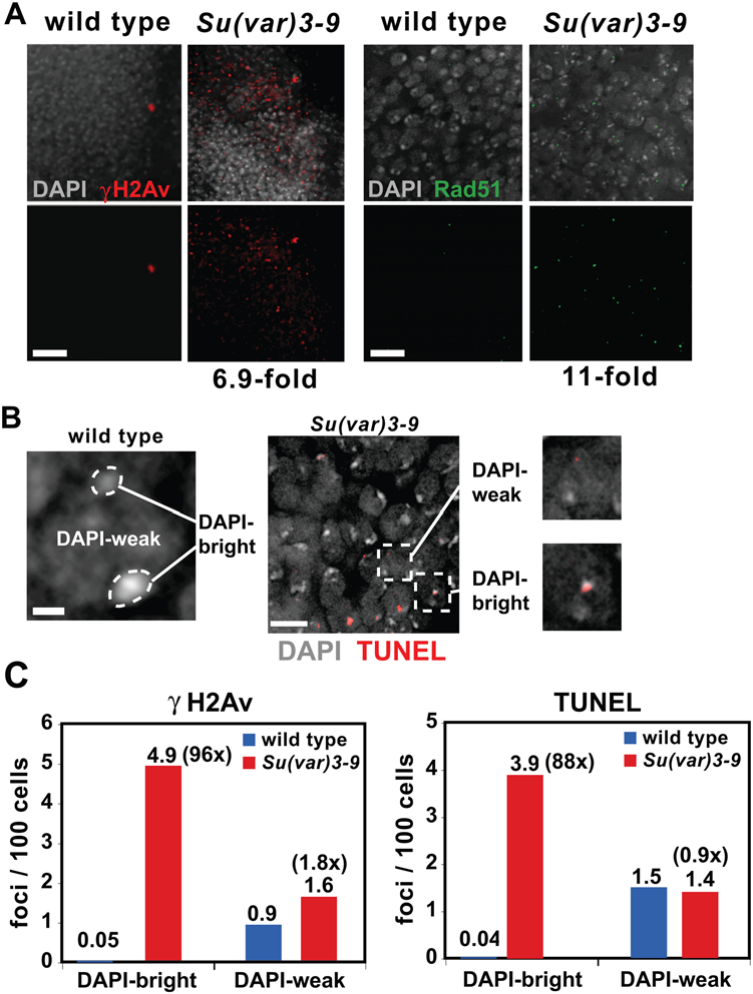
*Su(var)3-9* mutant somatic cells display increased DNA damage in heterochromatin. A) γH2Av (red) and Rad51 (green) IF in whole-mount diploid tissues from wild type and *Su(var)3-9* mutants are shown. Each image is an optical section. γH2Av- and Rad51-positive cells in *Su(var)3-9* are 96- and 11- fold increase over wild type. The p values were <0.01 by the Student's t test, and n>800 cells for each group. The scale bars = 25 mm in γH2Av IF images and 8 um in Rad51 images. B) An optical section shows a wild type diploid cell stained with DAPI; dashed lines encircle the DAPI-bright regions. Bar = 0.8 mm. Right panel are optical sections of *Su(var)3-9* mutant diploid cells stained by the TUNEL assay (red foci). The foci are double-stranded breaks recognized by TdT. Enlarged images showed examples of TUNEL foci in DAPI-weak and DAPI-bright regions. Bar = 4 mm. C) shows quantitative analysis of γH2Av and TUNEL signal localizations in wild type and *Su(var)3-9* cells. The distribution of γH2Av and TUNEL signals in DAPI-weak regions do not differ significantly between wild type and *Su(var)3-9* (p>0.05 by Chi-square test; n>40 for each genotype). Compared to wild type, γH2Av foci localized to DAPI-bright regions in *Su(var)3-9* is 96-fold higher and 88-fold for TUNEL (p<0.001 by Chi-square test; n>35 for each genotype).

Spontaneous DNA damage in whole-mount (three dimensional) larval brain and imaginal disc tissues from wild type and *Su(var)3-9* ^null^ mutants (generated from null mothers and fathers) was examined by indirect immunofluorescence (IF) with antibodies specific to γH2Av and Rad51 (Figure 1A). γH2Av is the phosphorylated form of the histone variant H2Av (at serine 137) and is associated with DNA repair sites [35]. The Rad51 protein facilitates repair of double-stranded breaks via homologous recombination [36]. We observed that *Su(var)3-9* somatic cells contained significantly increased frequencies of γH2Av and Rad51 foci in comparison to wild type (6.9-fold and 11-fold, respectively; p<0.001). Both γH2Av and Rad51 localization indicate sites of double-stranded breaks (DSBs), which we confirmed by the TUNEL (TdT-mediated dUTP nick end labeling) assay (Figure 1B; Materials and Methods).

H3K9me2 modifications are highly enriched in heterochromatin of *D. melanogaster*, suggesting that DNA damage associated with loss or mislocalization of this modification would be enriched in heterochromatin. Additionally, we had previously demonstrated that repeated DNA integrity is severely compromised in *Su(var)3-9* mutant cells, in terms of increased frequencies of extrachromosomal repeated DNAs [34]. Comparing damage frequencies in heterochromatin and euchromatin is challenging in this mutant, due to loss of the standard heterochromatin markers H3K9me2 and HP1. However, heterochromatic regions are associated with intense DAPI staining, due to the AT-rich nature of many heterochromatic sequences, and perhaps a higher degree of condensation. Therefore, we measured the colocalization of markers for DNA damage and repair with ‘DAPI-bright’ and ‘DAPI-weak’ regions of interphase cells (Figure 1B). This method of quantitation is conservative; it underestimates the amount of damage in heterochromatin, because some heterochromatic sequences are present in DAPI-weak regions.

γH2Av and TUNEL analysis showed that the numbers of foci localized in DAPI-weak regions do not significantly differ between wild type and *Su(var)3-9* cells (Figure 1C). In sharp contrast, foci localized to DAPI-bright regions in *Su(var)3-9* increased 96-fold over wild type for γH2Av and 88-fold for TUNEL (Figure 1C). IF analysis of *Su(var)3-9* demonstrated that 70% (±s.d. 7.9%) of foci in DAPI-bright regions contained both γH2Av and Rad51, which identifies *bona fide* DSBs, as opposed to other kinds of damage. We conclude that loss of Su(var)3-9 leads to increased DNA damage in somatic cells, and that most or all of the additional damage is DSBs in heterochromatin.

Reduced H3K9me in rDNA due to *Su(var)* mutations leads to formation of extrachromosomal ribosomal DNAs (rDNA) that can seed ectopic nucleolus formation [34]. Surprisingly, combined fluorescent in situ hybridization (FISH) and IF on whole-mount diploid tissues did not show significant colocalization between rDNA and γH2Av signals in the *Su(var)3-9* diploid cells (data not shown). This suggests that DNA breaks within rDNA are repaired and form eccDNA more efficiently than DSBs in other regions of heterochromatin.

### Increased DNA breaks occur in *Su(var)3-9* mutant oocytes and nurse cells

Classical genetic studies have shown that reciprocal meiotic recombination (crossing-over) occurs on average once per euchromatic arm per nucleus, but does not occur in heterochromatin [33],[37]. Previous studies showed that heterozygous combinations of *Su(var)* mutants results in reciprocal exchange in heterochromatin [38], suggesting that heterochromatin components repress recombination during meiosis.

We determined whether increased heterochromatic DNA damage and DSBs occur in *Su(var)3-9* mutant meiotic cells, as observed in somatic cells. The germarium is the part of *Drosophila* ovary that contains developing oocytes and nurse cells, which share the same cytoplasm. Although only the oocyte will progress through meiosis, both cell types express Spo11 (mei-W68 in *Drosophila*), which is required for generating DSBs during meiosis [33],[39].

**Figure 2:**
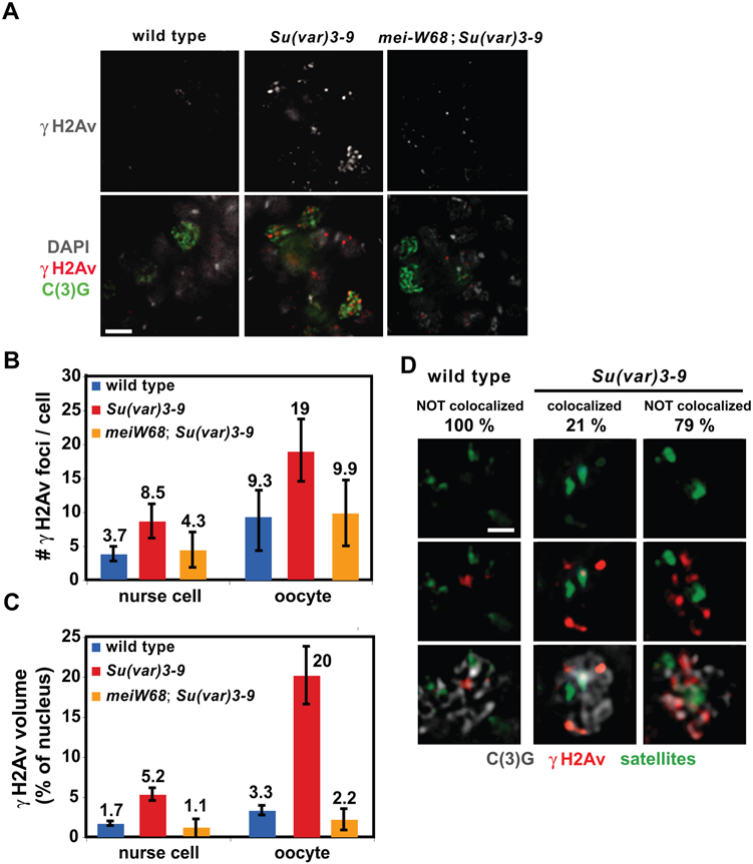
*Su(var)3-9* mutant oocytes and nurse cells display increased DNA damage in heterochromatin. A) The images show γH2Av (white in top panel and red in bottom panel) and C(3)G (green) IF in whole-mount germaria from wild type, *Su(var)3-9* and *mei-W68; Su(var)3-9*. C(3)G is part of the synaptonemal complex and used to distinguish oocytes from nurse cells, both of which contain DSBs. Each image is an optical section; bar = 7 mm. B) and C) The graphs show the average numbers and volumes (relative to total nuclear volumes) of γH2Av foci in nurse cells and oocytes from wild type, *Su(var)3-9* and *mei-W68*; *Su(var)3-9*. Both quantitation methods showed that γH2Av foci in *Su(var)3-9* nurse cells were significantly increased over wild type (p<0.001). γH2Av foci in *Su(var)3-9* oocytes were significantly increased over wild type (p<0.001). The numbers of γH2Av foci in *mei-W68*; *Su(var)3-9* nurse cells and oocytes were lower than *Su(var)3-9* alone and not significantly different from wild type (p<0.001). Error bars indicate standard deviations, p values were calculated by Student's t test, and n>15 for each cell type. D) Combined γH2Av IF (red) and satellite FISH (green) in wild-type and *Su(var)3-9* germaria; C(3)G (grey) staining identifies the oocytes. Percent of oocyte and nurse cells that displayed overlap between γH2Av and satellite signals are shown. Each image is an optical section, and cells are 5 mm wide.

IF analysis showed a dramatic increase in γH2Av signals in *Su(var)3-9* mutant germaria compared to wild type (Figure 2A). We performed two kinds of quantitative analyses because a high percentage of each nucleus in mutant cells stained for γH2Av. γH2Av foci can fuse with each other, and thus foci counts can under-represent the phenotypic severity in mutant cells. Quantitative volumetric analysis can address this issue, but can also be influenced by varying intensity values in whole-mount IF experiments. The number of γH2Av foci in *Su(var)3-9* nurse cells and oocytes were significantly increased over wild type (Figure 2B, p<0.001). Quantitative analysis of γH2Av volumes (relative to nuclear volumes) in wild type and *Su(var)3-9* yielded similar results (Figure 2C). Increased DNA damage foci in germaria may be due to higher frequencies of meiotic breaks or defects in repairing meiotic breaks. However, we did not observe any γH2Av signals in the *Su(var)3-9* late stage oocytes (data not shown), where meiotic crossover would have completed, suggesting that increased DNA breaks were not due to defective meiotic break repair in mutants.

Does the increased DNA damage in *Su(var)3-9* mutant oocytes and nurse cells occur in heterochromatin, as observed for somatic cells? The heterochromatic regions in oocytes and nurse cells do not coalesce into clearly-definable regions, and HP1 is mislocalized in *Su(var)3-9* cells due to severely reduced H3K9 methylation. Therefore, we performed combined γH2Av IF and FISH with satellite DNA probes in whole-mount wild type and *Su(var)3-9* mutant germaria. The probes included the 1.688, AACAC, AATAT, dodeca, AATAG, 1.686, and AAGAG satellites, which correspond to approximately 34 megabases of the heterochromatin (Materials and Methods), less than half of heterochromatic DNA. Nevertheless, γH2Av IF and satellite FISH signals overlapped in 21% (s.d. 9.6%, n = 151) of *Su(var)3-9* oocytes and nurse cells, and was never observed in wild type cells (n = 55, Figure 2D; p<0.001). These data show that a significant proportion of the elevated levels of DNA breaks in *Su(var)3-9* mutant oocytes and nurse cells occur in heterochromatin.

Elevated frequencies of DNA breaks in *Su(var)3-9* mutant ovaries could arise from increased access of heterochromatin to normal meiotic recombination pathways, which are mediated by Spo11, or by an independent mechanism, such as replication errors. We therefore examined the impact of meiW68 loss on repair foci formation in *Su(var)3-9* mutants. The number and volume of γH2Av foci in *mei-W68*; *Su(var)3-9* double mutant nurse cells and oocytes were significantly reduced in comparison to single *Su(var)3-9* mutant cells, and were not significantly different from levels observed in wild type cells (Figure 2A, B, and C). Therefore, Spo11/mei-W68 mediated events cause the majority of the increased DSBs in *Su(var)3-9* germ-line cells. The residual foci observed in the double mutants likely represent Spo11-independent breaks in response to eliminating Su(var)3-9.

Previous studies demonstrated that non-recombinant (achiasmate) chromosomes in *Drosophila* females are paired in the heterochromatin [40] and that heterochromatic homology is required for normal segregation [4]. We observed significant increases in the frequencies of 4^th^ and X chromosome exceptions (nondisjunction or loss) in *Su(var)3-9* mutant females compared to wild type females (Figure S1). These results demonstrate that Su(var)3-9 and H3K9 methylation are required for normal homolog segregation in female meiosis. Depletion of H3K9me2 could cause loss and nondisjunction due to defective heterochromatin-mediated pairing, sister chromatid cohesion, and/or high levels of recombination in heterochromatin [41].

We conclude that H3K9 methylation is important for maintaining the structural integrity of heterochromatin in meiotic as well as mitotic cells, and that most of the increased DSBs in *Su(var)3-9* meiotic cells arise through the canonical recombination pathway mediated by Spo11/mei-W68.

### *Su(var)3-9* mutant exhibits defective chromosome structures and increased loss of heterozygosity

**Figure 3:**
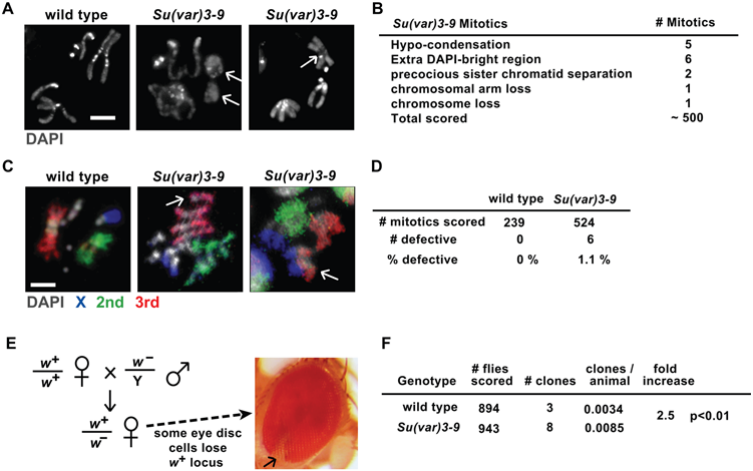
*Su(var)3-9* somatic cells exhibit genome instability phenotypes. A) DAPI staining of mitotic chromosomes from wild type and *Su(var)3-9* mutant diploid cells. Structural defects in *Su(var)3-9* mitotic chromosomes are indicated by white arrows. Each image is an optical section; bar = 2 mm. B) The chart shows quantitation of defective *Su(var)3-9* mitotic chromosomes. Some mitotic chromosomes exhibited more than one defect. C) Chromosome painting of mitotic chromosomes from wild type and *Su(var)3-9* mutant. Red = 3rd chromosomes, green = 2^nd^ chromosomes, and blue = X chromosomes. Fourth and Y chromosomes are only stained with DAPI. Structural defects, such as deletions and translocations, are indicated by white arrows. Each image is an optical section; bar = 2 mm. D) Quantitation showed that 1.1% of the *Su(var)3-9* mitotic chromosomes exhibited structural defects, compared to 0% for wild type (p<0.05 by Chi-square test). E) The diagram illustrates the genetic assay used to quantitate genome instability (loss of heterozygosity) in wild type and *Su(var)3-9* animals. Wild type virgin flies were mated with males hemizygous for *w* ^−^ (recessive *white* ^1118^ mutation) to produce females heterozygous for *w* ^−^ and *w* ^+^. A clone of *w−* adult eye cells (pictured) arises during larval development when the *w* ^+^ allele is lost due to mitotic recombination, deletion, or chromosome loss events. f) Quantitative analysis of *w−* clone frequencies in wild type and *Su(var)3-9* animals. The p values were calculated using the Chi-square test.

Persistent DNA damage could lead to chromosomal structural defects, rearrangements and aneuploidy. To test this hypothesis, we first examined wild type and *Su(var)3-9* mitotic chromosomes by DAPI-staining. All wild-type mitotic chromosomes exhibited banding patterns characteristic of heterochromatin (Figure 3A, first panel). In contrast, *Su(var)3-9* mitotic chromosomes exhibited a variety of phenotypes such as hypo-condensation (Figure 3A, second panel) and extra DAPI-bright bands (Figure 3A, third panel; complete list of phenotypic analyses is in Figure 3B).

We used FISH paints that hybridize to the euchromatic regions of three *Drosophila* chromosomes (X, 2 and 3; Materials and Methods) to determine if *Su(var)3-9* cells contain increased frequencies of rearranged chromosomes compared to wild type (Figure 3C). Quantitative analysis of the ‘painted’ chromosomes showed that 1.1% of *Su(var)3-9* mitotic chromosomes exhibit structural defects such as deletions, duplications, and translocations (Figure 3D), which were never observed in wild type.

We also determined if *Su(var)3-9* mutant animals exhibit increased loss of heterozygosity (LOH) compared to wild type. A genetic assay was performed to monitor the loss of *w* ^+^ from wild type and *Su(var)3-9* animals; loss of w+ in this assay could result from either chromosome loss or mitotic recombination (Figure 3E). We observed that *Su(var)3-9* animals spontaneously lose *w* ^+^ at a 2.5-fold higher frequency than in wild type (Figure 3F; p<0.01). Thus, *Su(var)3-9* animals exhibit hallmark characteristics of genome instability that can be observed cytologically and genetically.

### *Su(var)3-9* mutants are predominantly viable and fertile

**Figure 4:**
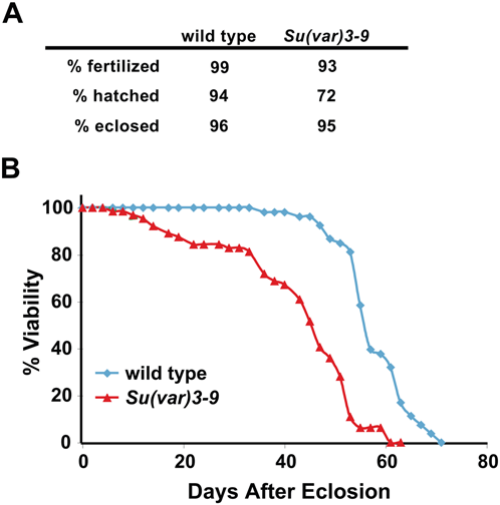
Developmental progression and lifespan of wild type and *Su(var)3-9* animals. A) Analysis of developmental progression for wild type and *Su(var)3-9* animals. Animals in the two groups laid comparable numbers of eggs. In all three assays, the p values comparing *Su(var)3-9* to wild type are <0.001 by Student's t test. n>150 for each genotype. B) The graph shows lifespan analysis of wild type and *Su(var)3-9* adult female flies. *Su(var)3-9* females displayed significantly shorter lifespan than wild type (p<0.01 by Wilcoxon signed rank test). n>50 female flies.

Previous analysis showed that mice deleted for both *Suv3-9* genes exhibit genome instability and partial prenatal lethality [42]. Surprisingly, despite elevated frequencies of DNA damage, chromosome rearrangements and LOH shown here, *Su(var)3-9* null mutant animals derived from null mothers are homozygous viable and fertile [43]. However, our analysis of survival at various developmental stages showed significant differences between wild type and mutant animals (Figure 4A). *Su(var)3-9* mutant parents produced 93% fertilized eggs, 72% of which hatch to embryos, compared to 94% hatching for wild type (Figure 4A). Defective development during embryogenesis is the likely cause of the lower hatch rates. Once they hatched into larvae, developmental timing and eclosion rates of *Su(var)3-9* mutants were comparable to wild type. Thus, *Su(var)3-9* mutant animals are mostly viable and fertile; larval and pupal development are normal, but they have elevated levels of unfertilized eggs and embryonic lethality compared to wild type. In addition, *Su(var)3-9* females exhibit significantly shorter adult lifespans compared to wild type (Figure 4B, p<0.01).

### DNA damage checkpoints are activated in *Su(var)3-9* cells

We hypothesized that *Su(var)*3-9 mutant animals are mostly viable and fertile [43] due to activation of checkpoints that delay cell cycle progression until DNA damage is repaired. To test this hypothesis, we compared the proportions of cells in different cell cycle stages in wild type and *Su(var)3-9* cells. IF was performed on squashed diploid cells using antibodies to PCNA (S phase), Cyclin A (CycA, S phase, G2 and mitosis), and PH3 (H3 phosphorylated at serine 10, mitosis); the TUNEL assay was performed to identify apoptotic cells, which contain labeling throughout the nucleus, as opposed to foci observed in response to DNA damage (Materials and Methods).

**Figure 5:**
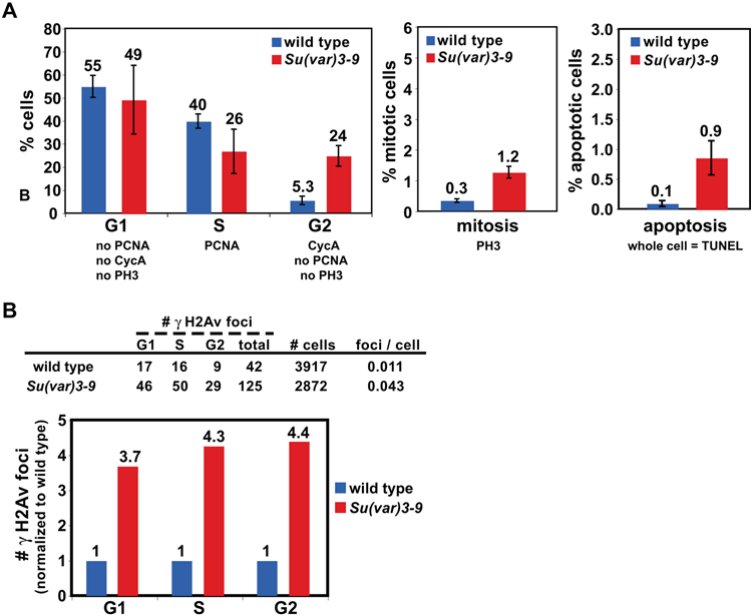
G2, mitotic, and apoptotic cell percentages are increased in *Su(var)3-9*. A) The histograms show cell cycle stage analysis of wild type and *Su(var)3-9* cells. The percent of G1 cells do not differ significantly (p>0.05). The percentage of S phase cells in *Su(var)3-9* is significantly lower than wild type (p<0.05). The percent of wild-type cells in G2 is significantly lower than in *Su(var)3-9*. Mitotic indicex in *Su(var)3-9* is 4-fold over wild type (p<0.001). The percent of apoptotic cells (whole nuclei contain TUNEL signals, instead of foci) in *Su(var)3-9* is 9-fold over wild type (p<0.001). P values were calculated by Student's t test, and n>1000 cells for each genotype. B) The chart shows γH2Av foci numbers in G1, S, and G2 cells of wild type and *Su(var)3-9*. Analysis of the ratios of γH2Av foci to total cell numbers during the cell cycle in wild type and *Su(var)3-9*. γH2Av foci in *Su(var)3-9* cells increased over wild type in G1, S, and G2 (p<0.01). P values were calculated by Chi-square test, and n>40 cells for each genotype.

Cells in G2 were identified by staining for CycA, but not PCNA or PH3, replicating cells were stained by PCNA, and G1 cells were not stained with CycA, PCNA, or PH3. Comparative analysis showed that the percentage of *Su(var)3-9* cells in S phase was lower than in wild type, and that the percentages of *Su(var)3-9* cells in G2, mitosis, and apoptosis increased relative to wild type (Figure 5A). These data suggest that G2 and mitotic checkpoints are activated in these mutant cells. Increased apoptosis in the mutant animals is likely caused by unrepaired DNA damage, which is mediated by the p53 pathway [28]. It is important to note that although the fold increases for mitotic and apoptotic cells in *Su(var)3-9* are large, the actual percent of cells are low (1 to 3%), in comparison to the 24% of cells in G2 (4.5 fold over wild type).

We next determined which cell cycle stages displayed spontaneous DNA damage in *Su(var)3-9* mutants. Comparisons of gH2Av foci with the cell cycle markers PCNA and CycA showed that DNA breaks are present in all interphase stages of the cell cycle in *Su(var)3-9* (Figure 5B and C). This assay cannot determine when DSBs occur, since they can persist from one cell cycle stage into another if they are not repaired. Nevertheless, the finding of DSBs in G1 strongly suggests that increased DNA breaks in heterochromatin of *Su(var)3-9* cells are not specific to DNA replication in S phase.

### DNA damage checkpoint pathways are critical for the viability of *Su(var)3-9* adults

**Figure 6:**
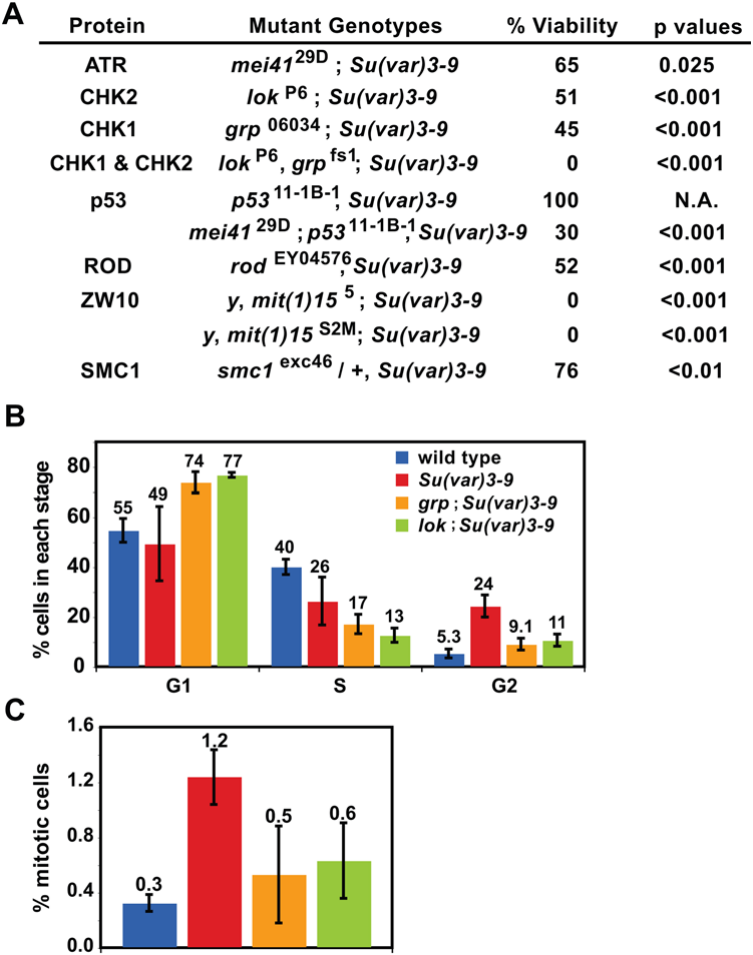
DNA repair checkpoint and mitotic checkpoint proteins are essential for the viability of *Su(var)3-9* mutants. A) The chart lists the viability of the double mutants of *Su(var)3-9* with mutations in the DNA damage checkpoint and mitotic checkpoint pathway. Viability was calculated relative to single homozygous checkpoint mutants, which are less viable than *Su(var)3-9* single mutants. Progeny counts are in Table S1. P values were calculated by the Chi-square test. B) Cell cycle analysis of wild type, *Su(var)3-9*, *grp*; *Su(var)3-9* and *lok; Su(var)3-9* mutant imaginal discs and brains. The percentages of G1 cells in the two double mutants were higher than wild type and single *Su(var)3-9* mutants. The percentages of S phase cells in the two double mutants are lower than wild type, but do not differ from *Su(var)3-9*. The percentages of G2 cells in the double mutants are lower than wild type and *Su(var)3-9*. The mitotic indices in the double mutants were lower than *Su(var)3-9* and do not significantly differ from the wild type. P<0.05 for all tests that show significant differences between wild type and *Su(var)3-9*; p values were calculated by Student's t test, and n>1000 cells for each genotype.

Increased proportions of G2 and mitotic cells in *Su(var)3-9* animals suggests that the G2 and mitotic cell cycle checkpoints may be activated by the increased frequencies of DSBs in heterochromatin. We thereby hypothesized that compromising the DNA damage checkpoint, using mutations in the checkpoint components, may result in lethality of *Su(var)3-9* animals. To test this hypothesis, we analyzed flies homozygous for *Su(var)3-9* and homozygous for mutations in DNA damage checkpoint activation (ATR/*mei-41*, Checkpoint kinase 1 (Chk1)/*grp*, and Checkpoint kinase 2 (Chk2)/*lok*). Animals double mutant for *Su(var)3-9* and cell cycle checkpoint mutations showed sub-viability ranging from 50% to 64.6% (Figure 6A; viability of double mutants were compared to single checkpoint mutants, which exhibit lower viability than *Su(var)3-9* mutants). *Su(var)3-9* mutant animals containing both *grp* and *lok* mutations are 100% lethal (Figure 6A). This demonstrates that DNA damage checkpoints are essential to the survival of *Su(var)3-9* animals. Cell cycle analysis showed that the percentage of *grp*; *Su(var)3-9* or *lok; Su(var)3-9* cells in S phase and G2 were lower than in *Su(var)3-9* alone, with a corresponding increase in cells in G1 (Figure 6B). Cell cycle characterization combined with the observed genetic interactions between *Su(var)3-9* and DNA damage checkpoint mutations demonstrate that the DNA damage checkpoint is activated in *Su(var)3-9* mutant animals, and is required for mutant viability.

We previously demonstrated significantly reduced levels of cohesin in heterochromatin in *Su(var)3-9* cells [34], which is likely to cause defects in sister chromatid cohesin and biorientation that result in an increased mitotic index. Indeed, reduction of cohesin levels by half (*smc1* heterozygotes) in *Su(var)3-9* animals reduced viability to 76.4% (Figure 6A). Chk1/*grp* and Chk2/*lok* have been shown to also regulate the metaphase-anaphase transition during mitosis [25],[26]. Our analysis showed that *grp* and *lok* mutations almost entirely suppress the mitotic index increase in *Su(var)3-9* mutants (Figure 6C). Mitotic checkpoint proteins Rod and ZW10 form a complex in the outer kinetochore, monitor microtubule attachments, and regulate the metaphase to anaphase transition [44]. *Su(var)3-9* and *rod* or *zw10* double mutants displayed synthetic lethality (51.7% and 0%, respectively, Figure 6A). The only available *rod* ^EY04576^ mutation is hypomorphic, and the *zw10* mutations are nulls, which could account for the viability differences in the double mutants. Regardless, these synthetic lethality data demonstrate that the mitotic checkpoint is also essential for *Su(var)3-9* survival.

*p53* ^11-1B-1^, *Su(var)3-9* double mutants exhibited 100% viability compared to *Su(var)3-9* single mutants (Figure 6A), suggesting that the apoptosis pathway, regulated by p53 in flies, does not impact the viability of *Su(var)3-9* animals. Even though apoptotic cells increase by 10-fold in *Su(var)3-9* mutants, they only account for 0.86% of cells (Figure 5A; s.d. 0.29%). We reasoned that the apoptotic pathway is only activated upon persistent DNA damage, which could produce the observed mitotic chromosomal defects (Figure 3). Indeed, triple mutant *mei-41*; *p53*, *Su(var)3-9* animals were less viable than *mei-41*; *Su(var)3-9* double mutants (Figure 6A). Thus, even though *p53* mutation (hence apoptosis) alone does not have significant impact, p53 does help ensure the viability of *Su(var)3-9* adults when the DNA damage checkpoint is compromised by *mei-41*/ATR mutations.

Are DNA repair factors also critical for the viability of *Su(var)3-9* animals? We observed that *lig4; Su(var)3-9* double mutants exhibited 100% viability (data not shown). However, the NHEJ pathway does not entirely depend on Ligase IV in *Drosophila* [45]. Such redundancy, in addition to functional compensations among different DNA repair pathways in *Drosophila* [19] make this viability analysis inconclusive.

### *dcr-2* mutant animals also exhibit increased DNA damage in heterochromatin

Heterochromatin formation and maintenance also requires the RNAi pathway, which can be subdivided into siRNA- and miRNA- based mechanisms. Dcr-2 is a critical component of the small-interfering RNA (siRNA) pathway in *D. melanogaster* [46], which regulates H3K9me2 localization to heterochromatin. We previously showed that H3K9me2 is mislocalized to a broader region of the nucleus in *dcr2* ^L811 fsx^ mutant cells, different from the strong reductions observed by IF in *Su(var)3-9* cells. However. *dcr2* mutants do contain significantly reduced levels of H3K9me2 at repeated DNAs, and significant increases in extrachromosomal repeated DNAs and ectopic nucleolus formation [34].

**Figure 7:**
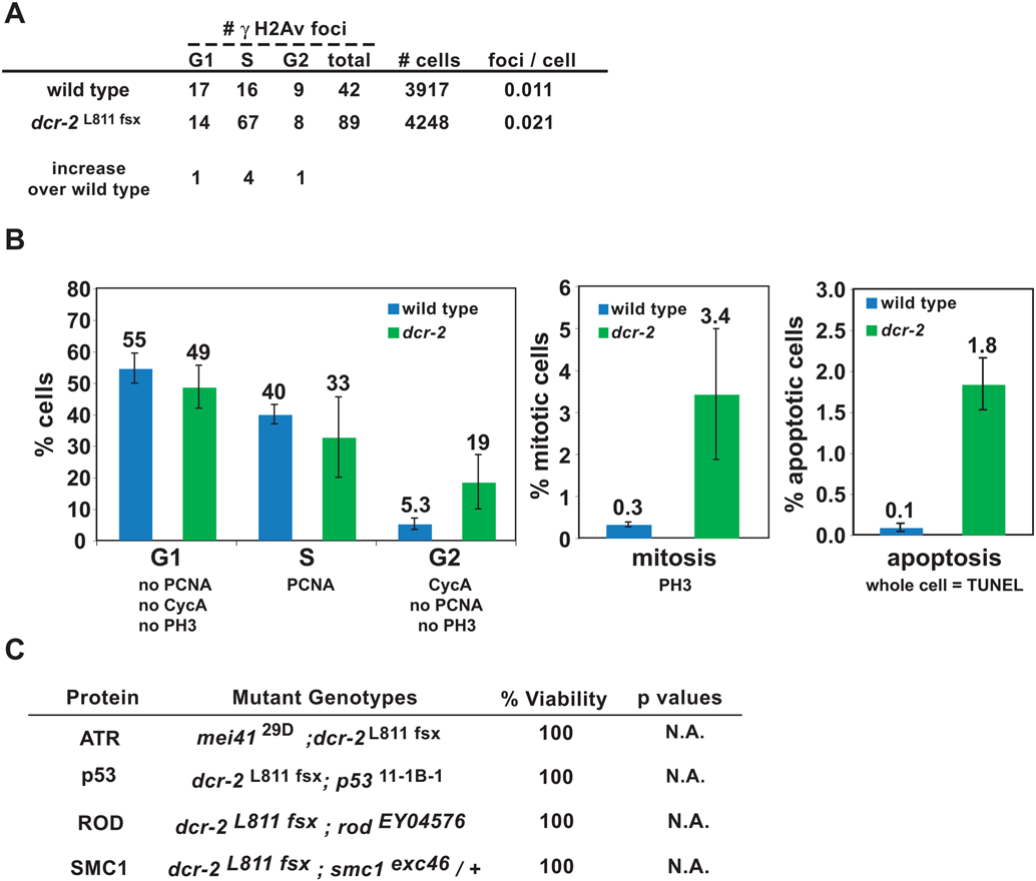
*dcr-2* mutants display increased spontaneous DNA damage in heterochromatin. A) Analysis of the ratios of γH2Av foci to total cell numbers at different cell cycle stages are shown for wild type and *dcr-2*. γH2Av foci in *dcr-2* cells only increased during S phase (p<0.05). P values were calculated by Chi-square test. B) The histograms show cell cycle stage analysis of wild type and *dcr-2* cells. The percent of G1 cells in the two groups do not differ significantly (p>0.05). The percentage of S phase cells is not significantly lower in *dcr-2* compared to wild type (p>0.05), but the percent of wild-type cells in G2 is significantly lower than in *dcr-2* (p<0.05). The mitotic index in *dcr-2* cells is 11-fold over wild type (p<0.001), and the percent of apoptotic cells (whole nuclei contain TUNEL signals, instead of foci) in *dcr-2* is 18-fold over wild type (p<0.001). P values were calculated by Student's t test, and n>1000 cells for each genotype. C) The chart lists the viability of double mutants of *dcr-2* with mutations in the DNA damage checkpoint and mitotic checkpoint pathways. Viability was calculated relative to single homozygous checkpoint mutants, which are less viable than *dcr-2* single mutants. Progeny counts are in Table S1. P values were calculated by the Chi-square test.

We have investigated whether patterns of DNA damage and repair are affected by loss of Dcr2, as reported here for *Su(var)3-9* mutants. Quantitative analyses of gH2Av and Rad51 foci in *dcr-2* mutant cells showed significant increases in spontaneous DNA damage and repair (2.1- and 3.5- fold over wild type, respectively; Figures S2A and B). Foci localized to DAPI-bright regions in *dcr-2* increased 24-fold over wild type for γH2Av and 33-fold for TUNEL (Figure S2C and D). Foci in DAPI-weak regions do not differ significantly between wild type and *dcr-2*. Therefore, increased DNA damage in *dcr-2* occurs in heterochromatic DNAs of *dcr-2* mutant cells. IF analyses showed that γH2Av foci in *dcr-2* mutant oocytes increased over wild type by 1.5- (foci number) to 3- fold (volumetric analysis). In both meiotic and mitotic cells, the increases in spontaneous DNA damage were significant, but less severe than in *Su(var)3-9* mutants (Figure S3). *Su(var)3-9* mutant cells contained elevated frequencies of damage in all interphase cell cycle stages (Figure 5B). However, gH2Av foci enrichment in *dcr-2* mutant cells only occurred during S phase (Figure 7A), suggesting that increased DNA breaks are repaired in S phase and do not persist into G2.

As observed for *Su(var)3-9*, *dcr-2* mutants displayed reduced embryonic viability, but developed normally in larval and pupal stages. The percentages of *dcr-2* cells in G2, mitosis, and apoptosis also increased relative to wild type (Figure 7B). However, viability analysis of double mutant flies showed that mutations in DNA damage or mitotic checkpoint proteins did not impact the viability of *dcr-2* mutants (Figure 7C). Thus, in contrast to the effects of *Su(var)3-9*, checkpoint activation is not necessary for the repair of endogenous DNA damage in *dcr-2* diploid cells, or for mutant viability.

It is possible that phenotypic differences in comparison to *Su(var)3-9* null mutants is due to the hypomorphic nature of the only available *dcr-2* allele [46], and retention of more H3K9me2 at heterochromatic sequences (Peng and Karpen, 2007). Nevertheless, these data demonstrated that compromising the siRNA pathway also leads to increased DNA damage in heterochromatin, reduced viability in embryogenesis, and activation of DNA damage checkpoints. We conclude that both the H3K9 methylation and siRNA pathways safeguard the integrity of heterochromatic DNA in meiotic and somatic cells, albeit with different levels of impact.

## Discussion

Genomes with complex DNA organization and high repeat content present challenges for maintenance of genome stability during DNA replication, repair, and recombination. Heterochromatin comprises approximately 30% of the *Drosophila* and human genomes, and ∼30% of human euchromatin is composed of transposons and other repeats [2],[47],[48]. Persistence of heterochromatin through evolution likely results from the many essential functions it encodes. We have demonstrated that maintaining the stability of heterochromatic DNA in mitotic and meiotic cells requires the siRNA and H3K9 methylation pathways. Increased spontaneous DNA damage in heterochromatin in *Su(var)3-9* and *dcr-2* mutants results from reduced H3K9me2 levels in heterochromatin, due to loss of the major H3K9 methyltransferase or misregulation of H3K9me2 localization, respectively [34]. Most or all of the increased DNA damage observed in mutants are located in heterochromatin, suggesting that the observed chromosomal defects and rearrangements and activation of DNA damage checkpoints are consequences of heterochromatin damage. The lower phenotypic severity exhibited by *dcr-2*, compared to *Su(var)3-9*, is correlated with the higher heterochromatic H3K9me2 content in *dcr-2* compared to *Su(var)3-9* mutants [34]. This phenotypic difference may be due to the hypomorphic nature of the *dcr-2* mutant [46].

A potential cause of increased damage to heterochromatic sequences in *Su(var)3-9* and *dcr-2* mutants is defective DNA replication. Replication of heterochromatin normally occurs in late S-phase [30], and analysis of PCNA staining revealed that the proportion of cells in S-phase in *Su(var)3-9* mutants is significantly lower than that in wild type. In the absence of H3K9 methylation, regions of repetitive DNA may be incompletely replicated or defective in chromatin reassembly [49] due to a shortened S phase. Alternatively, repeated DNA in heterochromatin may undergo faster replication, resulting in more errors; this is supported by the demonstration that heterochromatic regions are more efficiently endoreplicated in *Su(var)3-9* mutant polytene chromosomes [50]. Thus, H3K9 methylation may be required to delay replication in repetitive regions, allowing resolution of replication forks; DSBs would be produced by stalled forks and/or fork collapse in the absence of this control.

The demonstration that gH2Av foci were detected in G1, S and G2 stages in *Su(var)3-9* mutants suggests that defective DNA replication is not the only cause of the increased damage and repair foci in heterochromatin. Another explanation is that proper DNA damage detection and subsequent DNA repair response in heterochromatin may require H3K9me2. This model is supported by the recent demonstration that HP1β, whose localization requires H3K9me, is needed for efficient DNA damage detection in mammalian cells (Ayoub et al., 2008). This requirement for HP1 in the DNA damage response suggests that euchromatin (containing little H3K9me and HP1β) and heterochromatin likely exhibit different responses to DNA damaging agents. gH2A is not recruited to the silent HM region in *S. cerevisiae* when a DSB is generated in nearby euchromatin [51]. Studies of ionizing radiation followed by quantitation of DNA break frequencies over time indicated that the vast majority of DNA breaks are located outside the heterochromatin in interphase cells an hour after damage was induced [51],[52]. The lower frequencies of repair foci observed in heterochromatin suggests that euchromatin may be more prone to damage by ionizing radiation. Alternatively, initial damage frequencies within euchromatin and heterochromatin may be very similar, with faster repair of heterochromatic breaks. Experiments to differentiate between these two explanations are needed, such as comparing break frequencies within seconds/minutes of damage. In addition, analyses are required to determine if H3K9me chromatin at heterochromatic DNA is required for suppressing DSB formation by ensuring normal heterochromatic replication or protection from damaging agents, and/or proper function of the DNA damage response.

Mutations in the DNA damage checkpoint (*mei-41^29D^*, *grp^06034^*, and *lok^P6^*) exhibited synthetic lethality when combined with the *Su(var)3-9* mutations, further cementing the essential role of the DNA damage checkpoint for the viability of *Su(var)3-9* mutant animals. The incomplete synthetic lethality of the double mutants (∼50%) likely reflects redundancy of the checkpoint proteins; this is supported by the observation that mutations in both Chk1 and Chk2 cause complete lethality of *Su(var)3-9* mutants.

Intriguingly, *zw10* and *rod* mutations also exhibit synthetic lethality when combined with the *Su(var)3-9* mutations, suggesting that the Spindle Assembly Checkpoint (SAC) is also important for viability when H3K9me2 levels are reduced. Partial depletion of heterochromatic cohesin [34] and mitotic delays (this study) were observed in *Su(var)3-9* mutants, and *smc1/+*, *Su(var)3-9* double mutant animals displayed synthetic lethality. These observations suggest that reduced cohesion in *Su(var)3-9* mutants results in minor defects in bi-orientation and maintenance of spindle attachments, which normally do not affect cell or organismal viability due to SAC activation. However, when the SAC is abrogated by *zw10* or *rod* mutations, there is no mitotic delay and attachment defects cannot be fixed, resulting in missegregation and lethality. Mutations in Chk1 (*grp*) or Chk2 (*lok*) also suppress the increased mitotic index in *Su(var)3-9* cells. However, it is unclear at this time whether Chk1 and Chk2 are participating in an SAC response [25],[26], or are involved in a different checkpoint that is activated by DNA damage that persists into mitosis in these mutants.

The RNAi pathway directly impacts genome stability and the development of germlines in *D. melanogaster*, mammals, and *C. elegans*. Specifically, Piwi/Aubergine regulation of repeat associated small interfering RNAs (rasiRNAs) mediates silencing of retrotransposons and the repeated Stellate locus [53], and promotes normal embryonic axis specification and germline development [54]. Mutations in the rasiRNA pathway components, *armitage* and *aubergine* lead to disruption of embryonic axis specification and increased DNA breaks in meiotic cells [54]. In contrast to our observations, *mei-W68* mutations did not suppress DSB formation in rasiRNA mutants. Furthermore, increased DNA damage in *armitage* and *aubergine* is specific to the germline [54], and we did not observe microtubule disorganization in *Su(var)3-9* mutants (data not shown). Differences between the phenotypes in *Su(var)3-9*/*dcr-2* and rasiRNA mutants suggest that these pathways ensure genome integrity via different mechanisms.

The majority of studies of chromatin modifications focus on transcriptional regulation. Current epigenomic characterizations of different cancer types also put great emphasis on epigenetic regulation of transcription, showing dramatic chromatin alterations of oncogenes or tumor suppressor genes whose transcriptional deregulation contribute to cancer progression [55],[56]. Our findings contribute to the growing realization that local chromatin structure and epigenetic regulation also impact genome structural integrity (reviewed in [57]. We have shown that mutations affecting H3K9 methylation lead to increased spontaneous damage that is predominantly or exclusively located in heterochromatin. Consequences of this defect are chromosome structural defects and genome instability, events that are also correlated with uncontrolled cell growth and tumorigenesis. SUV39h double knockout mice exhibit partial embryonic lethality and genome instability [42], as well as global gene deregulation [58],[59]. It is therefore unclear if the impact of SUV39h on mammalian genome stability is due to a direct effect on DNA damage and repair in heterochromatin, or misregulation of key developmental genes and cell cycle regulators. In contrast, H3K9 methylation by Su(var)3-9 in *Drosophila* is restricted to heterochromatin (Langley and Karpen, unpublished). *Su(var)3-9* ^null^ mutations in *Drosophila* therefore present a unique opportunity to study the role of H3K9me chromatin in heterochromatin stability, with minimal indirect effects from transcriptional deregulation.

*Drosophila* heterochromatin, not euchromatin, resembles mammalian euchromatic genomes in their complex DNA organization. Mammalian systems may employ mechanisms similar to *Drosophila* heterochromatin to regulate the stability of repeated DNAs. Human Alu repeats and heterochromatin on human chromosome 1 (band 1q12) both contain ‘fragile’ sites associated with chromosomal rearrangements found in malignant cancers [60],[61]. Vulnerability of these DNA elements is also highly correlated with their chromatin composition [62],[63]. A more detailed understanding of how H3K9 methylation helps stabilize *Drosophila* heterochromatin would help direct efforts to elucidate how repeated DNAs are maintained in mammals.

## Materials and Methods

### Fly stocks

All fly stocks were raised at 22°C. We received the *grp* ^06034^, *rod^EY04576^*, *mit*(1)15^5^, and *p53* ^11-1B-1^ flies from the Bloomington stock center. The *lok* ^P6^ flies are from Michael Brodsky, *dcr2^L811fsx^* from Richard Carthew, *mit(1)15^S2M^* from Michael Goldberg, *smc1^exc461^* from Scott Hawley, *mei-W68^4572^* from Kim McKim, *Su(var)3-9* null alleles 6 and 17 from Gunter Reuter, *mei-41* ^29D^ from Tin Tin Su, and *lok^P6^*, *grp^fs1^* [64] flies from Michael Brodsky and Kent Golic. Fly crosses were performed using standard genetic techniques. *Su(var)3-9* ^null^ flies used in all experiments were transheterozygotes of alleles 6 and 17 produced from null (6/17) mothers, so they lacked both maternal and zygotic Su(var)3-9 protein. *dcr-2* mutant flies were also produced from homozygous mutant mothers. *rod* ^EY04576^ *Su(var)3-9* ^null^ flies, *p53* ^11-1B-1^ *Su(var)3-9* ^null^ flies, and *smc1* ^exc46^, *Su(var)3-9* ^17^ flies were made by meiotic recombination and scored by PCR reactions, using template DNA from single flies and primers that distinguish wild type from mutated DNA sequences.

### Antibodies

Rabbit antibodies that recognize γH2Av (1∶250 dilution) were purchased from Rockland (Gilbertsville, PA). The rabbit anti-Rad51 antibody (1∶100 dilution after direct labeling) was provided by Jim Kadonaga, and was directly labeled as previously described [65]. The mouse anti-C(3)G antibody (1∶500 dilution) was provided by Scott Hawley [66], and the rabbit anti-PCNA antibody (1∶100 dilution) was provided by Daryl Henderson [67]. Rabbit anti-PH3 (1∶1000 dilution) was purchased from Upstate (Charlottesville, VA). The anti-CycA mouse monoclonal antibody (1∶20 dilution) was purchased from the Developmental Studies Hybridoma Bank (Iowa City, IA). Alexa dye-conjugated secondary antibodies were purchased from Invitrogen (Carlsbad, CA) and used at 1∶500 dilution. Rhodamine-conjugated anti-DIG antibody was purchased from Jackson ImmunoResearch Laboratories (West Grove, PA) and used at a 1∶100 dilution.

### TUNEL assay

The TUNEL assay was performed in whole-mount tissues that were fixed with 4% paraformaldehyde in PBS and 0.2% of TritonX-100 (PBST), then washed in PBST and permeabilized overnight with PBST. Tissues were incubated with TUNEL buffer (1× TUNEL buffer from Roche, 2.5 mM CoCl2, 0.2% TritonX-100) for 10 min, then in TUNEL buffer, dNTPs (final concentrations of 10 uM of dATP, dCTP, and dGTP, 3.3 uM dTTP, and 6.6 uM DIG-dUTP) and TdT enzyme (20 U/ml final concentration; purchased from Roche Diagnostics (Mannheim, Germany) for 3 hours at 37°C. To analyze the percentage of cells in apoptosis, brain and imaginal disc tissues were squashed onto slides into single cell layer using standard techniques. The slides were washed extensively with PBST, incubated with TUNEL buffer, dNTPs and TdT enzyme for 2 hours at 37°C. After the TUNEL assay, DIG signals were detected via standard IF procedures using rhodamine-labeled anti-DIG antibody from Jackson ImmunoResearch (West Grove, Pennsylvania).

### Developmental stage analysis

#### % fertilization

Flies were allowed to lay eggs for 4 hours at 25°C on soft agar plates containing yeast paste. Eggs were incubated for 6 hours at 25°C, then fixed using standard methods [68]. Nuclei in the fixed eggs were visualized by DAPI staining. The percentages of fertilized eggs were calculated by the formula: ((total number of eggs – the number of eggs containing one or two nuclei)/total number of eggs)×100%.

#### % hatched eggs

Flies were allowed to lay eggs overnight at 25°C on soft agar plates containing yeast paste, and the numbers of eggs laid were counted. The eggs were allowed to incubate at 25°C for 30 hours, and the numbers of unhatched eggs were counted (which includes unfertilized eggs). The percentages of hatched eggs were calculated by the formula: ((number of eggs laid – number of unhatched eggs)/number of eggs laid)×100%.

#### % eclosion

Flies were allowed to lay eggs overnight in a bottle containing fly food at 25°C. The bottles were incubated at 25°C for 2 weeks. The eclosion percentages were calculated by the formula: (number of hatched pupa cases/total number of pupae)×100%.

### Lifespan analysis

More than 120 flies from each genotype, one day after eclosion, were separated into female and male populations, and passed onto new vials every other day and incubated at 25°C. Each vial contained approximately 20 flies. Dead flies were counted every other day. When all flies died, the total number of flies was summed from the numbers of dead flies. The viability percentages were calculated by dividing the number of flies alive at specific time periods by the total number of flies.

### IF, FISH, and IF-FISH of whole-mount tissues and squashed tissues

Whole mount IF was performed as previously described [34],[69]. Germaria were dissected within 24 hours of mating, fixed with 4% paraformaldehye in PBS, 0.3% Triton-X-100, and washed for 1 hour in PBS, 0.3% Triton-X-100. Germaria were permeabilized in PBS, 0.3% Triton-X-100 for 3 nights, blocked in PBS with 5% milk and 0.3% Triton-X-100. Fixed germaria were incubated in primary and secondary antibody solutions for >4 hours and washed for >1 hour. FISH was performed as previously described [5] using 100 ng of each probe. In combined IF-FISH experiments, tissues were fixed after IF then FISH analysis was performed. FISH probes targeting *Drosophila* satellite DNAs were made by 3′-end labeling of oligonucleotides (sequences are homologous to *Drosophila* satellite DNAs) with aminoallyl-dUTP (Sigma, St. Louis, MO) using the TdT enzyme, followed by conjugation to Alexa ester dyes (Invitrogen).

### Microscopy, volumetric, and colocalization analysis

All images were captured using an Applied Precision Deltavision microscope (Issaquah, Washington) and deconvolved by the SoftWorx software (also from Applied Precision), using the conservative algorithm with 5 to 8 iterations. The SoftWorx-deconvolved images were converted to TIFF files and then into image stacks for volumetric analysis with the Metamorph 7.0 software (Molecular Devices; Downingtown, PA). The Metamorph 7.0 volumetric analysis application identified individual foci within the image stacks, and the foci were then manually counted.

For foci localization and colocalization studies, optical sections of deconvolved images were enhanced for contrast and counted with respect to localization to DAPI-bright versus DAPI-weak regions. DAPI signals were not enhanced contrast. DAPI-bright regions were regions that contain contiguous (>5 pixels) bright DAPI signals; representative DAPI images are shown in Figure 1C.

Statistical comparisons and p values were calculated using the Chi-square test or Student's t test, assuming two-sample tails and unequal variance.

### Chromosome paints

FISH chromosome paints were made by degenerate PCR. The PCR products were digested with 4-base restriction enzymes, AluI, HaeIII, MseI, MspI, RsaI, and Sau3AI. Digested DNAs were end-labeled with TdT using aminoallyl-dUTPs followed by dye conjugation. Templates for the chromosome 2 and 3 paints were provided by Aki Minoda and Roger Hoskins, and were composed of genome tiling-path BACS identified by the Berkeley Drosophila Genome Project as low in repeat content and spaced ∼500 kb apart. Templates for the X chromosome paints were provided by Abby Dernburg, who micro-dissected polytene X chromosomes and amplified them via degenerate PCR [70].

FISH using chromosome paints were performed as follows. Acid-squashed preparations were treated with an ethanol series (incubation for 2 minutes each in 70%, 85%, and 95% ethanol at room temperature), incubated in 0.005% pepsin in 10 mM HCl for 1 minute, rinsed in PBS, and treated with an ethanol series to dry. The slides were treated with 2× SSCT (0.1% Tween-20) for 5 minutes, 50% formamide in 2× SSCT for 5 minutes, and 70% formamide in 2× SSCT for 5 minutes. Chromosomes on slides, incubated in 70% formamide and 2× SSCT, were denatured on the heat block of a PCR machine programmed to increase the temperature from 25 to 74°C within 1.5 minutes, stay at 74°C for 1.5 minutes, and decrease the temperature from 74 to 25°C within 1.5 minutes. The slides were dried with an ethanol series, and the denatured probes (in 50% formamide, 10% dextran sulfate, 2× SSCT, 1 ug Cot-1 DNA) were added to chromosomes and hybridized overnight. After the incubation, the coverslips were removed, and the slides were washed with 50% formamide, 2× SSCT at 37°C for 4 times, for 30 minutes each time.

### Mosaic eye and genome instability assay

Described in the Figure 3D legend.

### Cell Cycle analysis

Brain and imaginal discs were fixed in 4% paraformaldehyde and PBS for 5 minutes, then washed with PBS 4 times for 5 minutes each. The fixed tissues were incubated in Collagenase solution (0.04% Collagenase type IV, Sigma, in PBS) for 10 minutes, squashed onto slides using RainX-treated coverslips, and frozen in liquid nitrogen. After coverslips were removed, the slides were allowed to warm for less than 30 seconds, fixed in 4% paraformaldehyde and PBS for 5 minutes, and washed with PBS 4 times for 5 minutes each. IF with cell cycle markers were performed using methods described [71], except no TritonX100 was used for CycA IF. Images were captured using an Applied Precision Deltavision Workstation and converted to TIFF files. The multi-wavelength cell scoring application within Metamorph 7.0 software was used to score cells positive for cell cycle markers or TUNEL signals. For each genotype and each marker, >3000 cells from at least 3 animals were analyzed.

## Supporting Information

Figure S1 Effects of *Su(var)3-9* on chromosome segregation in female meiosis. Nonrecombinant chromosomes bypass the normal requirement for chiasma formation by using the ‘achiasmate segregation system.’ 4^th^ chromosomes are always nonrecombinant (achiasmate), and 5% of normal sequence X chromosomes (*y*/*y*) are nonrecombinant. The frequency of non-recombinant X chromosomes is increased to 100% in *FM7* (balancer)/*y* heterozygotes. The following crosses were performed to monitor X and 4^th^ chromosome segregation simultaneously in wild type and *Su(var)3-9* null females, with and without suppression of X recombination (*FM7*/*y* and *y*/*y*, respectively): *FMY*/y; *Su(var)3-9* ^null^; *spa* ^pol^ X y+/Y ; *C(4)ci ey*/0 (53 females, 2035 progeny). *FMY*/y; *ry* ; *spa* ^pol^ X y+/Y ; *C(4)ci ey*/0 (89 females, 4226 progeny). *y*/*y*; *Su(var)3-9* ^null^; *spa* ^pol^ X y+/Y; *C(4)ci ey*/0 (27 females, 1093 progeny). *y*/*y*; *ry*; *spa* ^pol^ X y+/Y; *C(4)ci ey*/0 (49 females, 2391 progeny). Frequencies of total exceptions, which includes both loss and nondisjunction (ND) events, were all calculated using the methods described previously [72]. The frequencies of meiotic exceptions increased in *Su(var)3-9* mutant females compared to wild type most dramatically for the X chromosome (17 fold for *FM7*/*y*, 25 fold for *y*/*y*), but also for the 4^th^ chromosome (4.6 fold for *FM7*/*y*, 3.3 fold for *y*/*y*). 4^th^ chromosome ND, rather than loss, was increased in the mutants, suggesting defects in achiasmate homolog pairing, as opposed to cohesion or spindle attachment. X chromosome loss increased dramatically in both *FM7*/*y* and *y*/*y* females, but X ND frequencies only increased when X recombination was suppressed. The observation that X exceptions increased even in the absence of an X chromosome balancer (*y*/*y* females) suggests that segregation of both recombinant and non-recombinant chromosomes are affected by reduced H3K9 methylation. Previous studies showed that chromosomes with very proximal recombination events are more prone to ND and loss [41]; thus, the observed increase in heterochromatic DSBs in *Su(var)3-9* oocytes (Figure 2) is the most likely cause of the increased ND and loss.
(1.04 MB EPS)

Figure S2 *dcr-2* mutant somatic cells also display increased DNA damage in heterochromatin. A) and B) γH2Av (red) and Rad51 (green) IF in whole-mount diploid tissues from wild type and *dcr-2* mutants are shown. Each image is an optical section. γH2Av- and Rad51-positive cells in *Su(var)3-9* are 2.1- and 3.5- fold increase over wild type. The p values were <0.01 by the Student's t test, and n>800 cells for each group. The scale bars = 25 mm in γH2Av IF images and 8 um in Rad51 images. C) and D) show quantitative analysis of γH2Av and TUNEL signal localizations in wild type and *dcr-2* cells. The distribution of γH2Av and TUNEL signals in DAPI-weak regions do not differ significantly between wild type and *dcr-2* (p>0.05 by Chi-square test; n>40 for each genotype). Compared to wild type, γH2Av foci localized to DAPI-bright regions in *dcr-2* is 24-fold higher and 29-fold for TUNEL (p<0.001 by Chi-square test; n>35 for each genotype).
(2.74 MB EPS)

Figure S3 *dcr-2* mutant oocytes display increased DNA damage in heterochromatin. A) The images show γH2Av (white in top panel and red in bottom panel) and C(3)G (green) IF in whole-mount germaria from wild type and *dcr-2* mutant. C(3)G is part of the synaptonemal complex and used to distinguish oocytes from nurse cells, both of which contain DSBs. Each image is an optical section; bar = 7 mm. B) and C) The graphs show the average numbers and volumes (relative to total nuclear volumes) of γH2Av foci in nurse cells and oocytes from wild type and *dcr-2*. Both quantitation methods showed that γH2Av foci in *dcr-2* oocytes were significantly increased over wild type (p<0.01), while foci in mutant nurse cells do not. Error bars indicate standard deviations, p values were calculated by Student's t test, and n>15 for each cell type.
(7.17 MB EPS)

Table S1 Progeny counts for genetic crosses used to calculate the viability of single and double mutants.
(0.02 MB XLS)
